# Comparative Proteomic Profiling of Clinical *Klebsiella pneumoniae* Strains and the Corresponding Outer Membrane Vesicles

**DOI:** 10.1002/jex2.70135

**Published:** 2026-04-14

**Authors:** Zongping Li, Jingyuan Xi, Xinmiao Jia, Yangzhige He, Guibin Wang, Shiyu Chen, Xiaobing Chu, Qian Zhang, Ying Zhu, Wei Yu, Peiyao Jia, Xiaoyu Liu, Qiwen Yang

**Affiliations:** ^1^ Department of Clinical Laboratory, State Key Laboratory of Complex Severe and Rare Diseases Peking Union Medical College Hospital Chinese Academy of Medical Sciences and Peking Union Medical College Beijing China; ^2^ Graduate School Peking Union Medical College, Chinese Academy of Medical Sciences Beijing China; ^3^ Department of Clinical Laboratory Center Beijing Children's Hospital, Capital Medical University, National Center for Children's Health Beijing China; ^4^ Center for bioinformatics, National Infrastructures for Translational Medicine Institute of Clinical Medicine & Peking Union Medical College Hospital, Chinese Academy of Medical Sciences and Peking Union Medical College Beijing China; ^5^ State Key Laboratory of Medical Proteomics, Beijing Proteome Research Center, National Center for Protein Sciences(Beijing) Beijing Institute of Lifeomics Beijing China; ^6^ Key Laboratory of Pathogen Infection Prevention and Control (Peking Union Medical College) Ministry of Education Beijing China

**Keywords:** antimicrobial resistance, comparative proteomics, hypervirulence, *klebsiella pneumoniae*, outer membrane vesicle, protein module

## Abstract

*Klebsiella pneumoniae (K. pneumoniae)* has become a global threat due to the convergence of carbapenem resistance and hypervirulence. Outer membrane vesicles (OMVs) play important roles in bacterial pathogenicity, yet their functional coordination with strains remains unclear. Here, we performed comparative proteomic profiling of six representative groups of *K. pneumoniae*, covering four major phenotypes, including carbapenem‐resistant hypervirulent (CR‐hvKP), represented by ST11‐K47 and ST11‐K64, carbapenem‐susceptible hypervirulent (CS‐hvKP), represented by ST23‐K1 and ST65‐K2, carbapenem‐resistant classical (CR‐cKP), and carbapenem‐susceptible classical (CS‐cKP). Integration of weighted co‐expression network analysis, pathway enrichment, and protein‐protein interaction analysis revealed distinct proteomic organisation and functional profiles between strains and OMVs. CR‐hvKP strains exhibited high cellular expression of resistance and DNA repair proteins. CS‐hvKP (ST23‐K1) showed elevated capsule biosynthesis proteins in strains and biofilm‐associated proteins in OMVs, whereas CS‐hvKP (ST65‐K2) strains packaged two‐component system proteins into OMVs. Module preservation and correlation analyses demonstrated limited preservation and weak correlation between cellular and OMV proteomes, suggesting that OMV protein organisation is largely distinct from the cellular proteome. Our findings highlight the specialised roles of OMVs, providing new insights into the pathogenic mechanisms of *K. pneumoniae* and potential therapeutic targets.

## 1 Introduction

*Klebsiella pneumoniae (K. pneumoniae)*, an opportunistic Gram‐negative pathogen, is a major cause of diverse clinical infections ranging from pneumonia and urinary tract infections to life‐threatening liver abscesses and sepsis (Paczosa and Mecsas 2016; Antimicrobial Resistance Collaborators 2022). In recent years, the convergence of multidrug resistance (MDR) and hypervirulence has given rise to high‐risk *K. pneumoniae* lineages that pose severe challenges to public health (Morris and Cerceo 2020; Dalton et al. 2020). In China, carbapenem‐resistant *K. pneumoniae* (CRKP), particularly the ST11 lineage with capsular serotypes K47 and K64, is the predominant clone in hospital settings and is associated with poor clinical outcomes (Wei et al. 2022). Meanwhile, hypervirulent *K. pneumoniae* (HvKP), exemplified by clones such as ST23‐K1 and ST65‐K2, continues to spread in community and hospital environments (Liao et al. 2020; Zhang et al. 2017; Wyres et al. 2020; Ye et al. 2016). The simultaneous rise of resistant and hypervirulent strains (CR‐hvKP) has created a dual threat to public health, elevating both treatment failure rates and mortality (Huang et al. 2025; Zhang et al. 2024). However, the distinct molecular features between these clinically important lineages remain largely unelucidated.

An increasingly recognised yet underexplored aspect of *K. pneumoniae* pathogenicity involves outer membrane vesicles (OMVs), nanoscale bilayered vesicles mainly released by Gram‐negative bacteria (Lucena et al. 2023). Accumulating evidence suggests that OMVs can act as delivery vehicles for virulence factors (VFs) and antimicrobial resistance proteins (AMRs), playing diverse roles in bacterial communication, biofilm formation, immune modulation, and horizontal gene transfer (Hu et al. 2024; Wang et al. 2022; Tao et al. 2025).

Although several studies have characterised the proteome of *K. pneumoniae* OMVs, most were conducted under stress conditions, such as antibiotic or phage exposure, focusing on stress responses rather than the baseline state (Mao et al. 2024; Hussein et al. 2023; Queiroz et al. 2021). Moreover, existing work typically examines either whole‐cell (Hao et al. 2022; Wiśniewski et al. 2009) or OMV proteomes in isolation, leaving their functional interplay largely unaddressed. How strains and their OMVs coordinate to mediate pathogenicity thus remains unclear. Clarifying the similarities and differences between these proteomic profiles is essential for understanding the contribution of OMVs to antimicrobial resistance, virulence, and the adaptive strategies of *K. pneumoniae*.

To bridge this critical knowledge gap, we performed a comparative proteomic profiling of strains and OMVs across six representative groups of *K. pneumoniae*, covering four major phenotypes, including carbapenem‐resistant hypervirulent (CR‐hvKP), represented by ST11‐K47 and ST11‐K64; carbapenem‐susceptible hypervirulent (CS‐hvKP), represented by ST23‐K1 and ST65‐K2; carbapenem‐resistant classical (CR‐cKP) and carbapenem‐susceptible classical (CS‐cKP). Specifically, we aimed to (1) identify specific proteomic signatures at both cellular and OMV levels; (2) decipher the functional similarity and divergence between strains and OMVs in supporting resistance, virulence, and overall fitness.

## 2 Materials and Methods

### 2.1 Sample Collection

*K. pneumoniae* isolates were collected from 19 hospitals participating in national antimicrobial surveillance programs, including the Antimicrobial Testing Leadership and Surveillance (ATLAS), the Study for Monitoring Antimicrobial Resistance Trends (SMART), and the China Bloodstream Gram‐negative Pathogens Antimicrobial Resistance and Virulence Surveillance Network (CARVIS‐NET). Based on carbapenem resistance genes, major virulence determinants, multilocus sequence typing (ST), and capsular serotype (K‐locus), the strains were classified into six groups, including CR‐hvKP (ST11‐K47), CR‐hvKP (ST11‐K64), CS‐hvKP (ST23‐K1), CS‐hvKP (ST65‐K2), CR‐cKP, and CS‐cKP. In total, 30 representative isolates (five per group, *n* = 5) were included, and for each isolate, proteomic analyses were performed on both the strains and the corresponding OMVs.

### 2.2 Bacteria Culture and OMVs Isolation

Bacterial stocks were stored at −80°C. After thawing, they were inoculated and streaked onto China Blue agar plates for isolation. A single isolated colony was inoculated into 1 mL Luria‐Bertani (LB) broth and grown in a shaking incubator (37°C, 220 rpm) until reaching 1.0 McFarland. The culture was then diluted into 100 mL fresh LB broth and incubated at 37°C, 220 rpm for 12 h. Afterward, cultures of bacteria were centrifuged at 4000 × *g* for 30 min. The supernatant was filtered through a 0.22 µm pore‐size sterile filter to remove cell debris and then ultracentrifuged for 70 min at 100,000 × *g* to pellet OMVs and resuspended in PBS to ultracentrifuged again. All centrifugation steps were performed at 4°C (Wiśniewski et al. 2009).

### 2.3 Protein Extraction

Each sample was supplemented with 200 µL of lysis buffer containing 8 M urea and protease inhibitors. The samples were thoroughly lysed and mechanically disrupted, followed by ultrasonication for 3 min. Subsequently, the lysates were centrifuged at 15,000 × *g* for 10 min to remove debris. The protein concentration in the supernatants was determined using the BCA protein assay kit.

### 2.4 Enzymatic Digestion and Desalting

To reduce disulphide bonds, 10 mM dithiothreitol (DTT) was added to each protein extract, and the samples were incubated at 56°C for 1 h. After cooling to room temperature, 55 mM iodoacetamide (IAA) was added for alkylation, and the samples were incubated in the dark at room temperature for 45 min. Based on the measured protein concentrations, appropriate amounts of protein were subjected to enzymatic digestion using the filter‐aided sample preparation (FASP) method (Wiśniewski et al. 2009). On the following day, the ultrafiltration tubes were centrifuged at 13,000 × *g* for 10 min at room temperature. The filtrates were transferred to new microcentrifuge tubes and dried under vacuum. Finally, the digested peptides were desalted using C18 desalting columns.

### 2.5 LC‐MS/MS Analysis

Lyophilised peptide samples were reconstituted in 0.1% formic acid (FA). For strains, samples were centrifuged at 17,000 g for 15 min, and the supernatant was loaded using an EASY‐nLC 1200 system (Thermo Scientific) equipped with a pre‐column (150 µm inner diameter, 1.9 µm particle size) under 400 bar pressure. Peptides were eluted with a 90‐min linear gradient from 6% to 30% solution B (95% ACN, 0.1% FA). Mass spectrometry was performed on a Thermo Orbitrap Q Exactive HF instrument. Full MS scans were acquired at 60,000 resolution (m/z 300–1500), followed by data‐dependent MS/MS scans at 15,000 resolution using HCD fragmentation (normalised collision energy 28). For OMVs, reconstituted peptides were centrifuged at 15,000 g for 15 min, and 500 ng of peptides per sample were separated on an EASY‐nLC 1200 system coupled to a custom‐packed analytical column (75 µm × 20 cm, 1.9 µm) at a flow rate of 300 nL/min. The gradient was set as follows: 6%–30% B over 42 min, 30%–42% B from 42–51 min, increasing to 95% B in 5 min and holding until 60 min. Mass spectrometric analysis was conducted on a Thermo Q Exactive HF instrument using data‐independent acquisition (DIA) parameters: full MS scans at 120,000 resolution (m/z 398–900) and MS/MS scans at 30,000 resolution with 32 isolation windows.

### 2.6 Database Search and Protein Identification

Raw data from strains were processed using Proteome Discoverer 2.4 (Thermo Fisher Scientific). Searches were performed against a composite database containing the UniProt *(K. pneumoniae)* subsp. pneumoniae HS11286 reference proteome, the Comprehensive Antibiotic Resistance Database (CARD), and the Virulence Factors Database (VFDB). Trypsin digestion was specified with up to two missed cleavages. Precursor and fragment mass tolerances were set to 15 ppm and 0.05 Da, respectively. Variable modifications included methionine oxidation and protein N‐terminal acetylation. The false discovery rate (FDR) was controlled at < 1.0% at both peptide and protein levels, requiring at least one unique peptide for protein identification. Raw data from OMVs were analysed using Spectronaut 18 (Biognosys) against the same composite database. Search parameters included trypsin digestion with up to two missed cleavages, fixed carbamidomethylation of cysteine, and variable modifications of methionine oxidation and N‐terminal acetylation. The FDR for peptide and protein identification was set to < 1.0%, with a requirement of at least one unique peptide per protein.

### 2.7 Nanoparticle Tracking Analysis (NTA)

NTA was performed to determine the size and concentration of the OMVs using a NanoSight NS300 instrument (Malvern, UK). Standard measurement was adjusted. The precipitated EVs were evenly dissolved in PBS and then diluted to a concentration between 1 × 10^7^ particles/mL and 1 × 10^9^ particles/mL, which were loaded into a 1 mL syringe. The samples were injected into the sample cell at a syringe speed of 40 µL/s. Sample videos were analysed using NanoSight Software NTA3.2 (Li et al. 2025). As part of a methodological pilot study prior to the main proteomic analysis, one representative OMV sample was used for NTA to validate the isolation protocol and characterise vesicle size distribution.

### 2.8 Transmission Electron Microscopy (TEM)

For the negative staining, 10 µL purified OMVs were applied to a carbon‐coated grid and incubated for 3 min. The grid was then washed with water and stained with 2% uranylacetate for 2 min and then examined by a transmission electron microscope (JEOL, Tokyo, Japan) at 80 kV (Xi et al. 2023). Similarly, TEM imaging was performed on a pilot OMV sample to confirm the presence of bilayer vesicles and assess morphological integrity before proceeding with proteomic profiling.

### 2.9 Proteomic Data Preprocessing and Statistical Analyses

Prior to downstream analysis, proteins identified by fewer than two unique peptides were excluded to minimise the contribution of low‐confidence identifications. Proteins detected (non‐zero intensity) in at least 80% of one group were retained. Proteomic data (strains and OMVs) were log2‐transformed and normalised using median normalisation. Missing values were imputed using the MissForest algorithm.

Virulence factors and antimicrobial resistance factors were referenced in the Virulence Factor Database (VFDB, https://www.mgc.ac.cn/VFs/) and the Comprehensive Antibiotic Resistance Database (CARD, https://card.mcmaster.ca/), respectively. Differentially expressed proteins were identified using the limma package (v3.60.6) in R, with the selection criteria set to |log_2_FC| > 0.585 and adjusted *p*‐value < 0.05. Protein–protein interaction (PPI) networks of differentially expressed proteins (DEPs) were constructed using the STRING database (https://string‐db.org/) with a confidence score > 0.4 and visualised with Cytoscape software (v3.9.1) (Van Parys et al. 2017). KEGG ontology was identified with eggNOG‐mapper (v.2.1.921). The subcellular location of proteins was predicted by the PSORTb online tool (v.3.0.3). Proteomic datasets from strains and OMVs were processed separately using R (v. 4.4.2). For downstream analysis, isolates were grouped into six phenotypic groups (*n* = 5 per group). All statistical comparisons, including differential expression analysis and WGCNA, were performed within each compartment (strains or OMVs) using group‐level biological replicates.

### 2.10 Weighted Co‐Expression Network Analysis

Proteomic co‐expression networks were independently constructed for the strains dataset and OMV dataset using weighted gene co‐expression network analysis (WGCNA) as previously described (Zheng et al. 2025; Johnson et al. 2022; Huang et al. 2023). Hierarchical clustering was performed to assess sample quality, removing one outlier from each dataset. The soft‐thresholding power (β) was determined using the pickSoftThreshold function, targeting an approximate scale‐free topology with a fitting index (*R* ^2^) above 0.8 and a slope close to −1. A β value of 5 was selected for the strains dataset, and a value of 8 was selected for the OMV dataset.

Signed adjacency matrices were subsequently transformed into topological overlap matrices (TOMs), and average linkage hierarchical clustering was applied. Modules were identified using dynamic tree cutting with a minimum module size of 30 and were merged based on a height cutoff of 0.25. Module Eigenproteins were then correlated with *K. pneumoniae* groups, including CR‐hvKP (ST11‐K47), CR‐hvKP (ST11‐K64), CS‐hvKP (ST23‐K1), CS‐hvKP (ST65‐K2), CR‐cKP, and CS‐cKP. Modules with *p*‐values less than 0.05 were considered statistically significant.

To assess the internal robustness of network construction, a split‐sample module preservation analysis was further performed separately for the strains and OMV datasets. Within each compartment, samples were randomly divided into a reference subset (70%) and a test subset (30%) using stratified sampling according to phenotypic group labels. Module preservation statistics were calculated using the modulePreservation function in the WGCNA R package with 200 permutations. Preservation was assessed using Zsummary. A Zsummary > 10 was considered strong preservation, 2< Zsummary ≤ 10 indicated weak to moderate preservation, and Zsummary ≤ 2 indicated no evidence of preservation. This analysis was used to evaluate within‐dataset stability of the strain and OMV co‐expression networks under sample resampling.

### 2.11 Cross‐Compartment Module Preservation Analysis

To compare the network organisation between cellular and OMV proteomes, cross‐compartment module preservation analysis was performed using the modulePreservation function in the WGCNA R package with 200 permutations. Preservation statistics were calculated by testing OMV modules in the strain dataset. The Zsummary score was used to quantify the degree of preservation, applying the same thresholds described in Section 2.10.

## 3 Results

### 3.1 Proteomic Overview of Strains and OMVs

**FIGURE 1:**
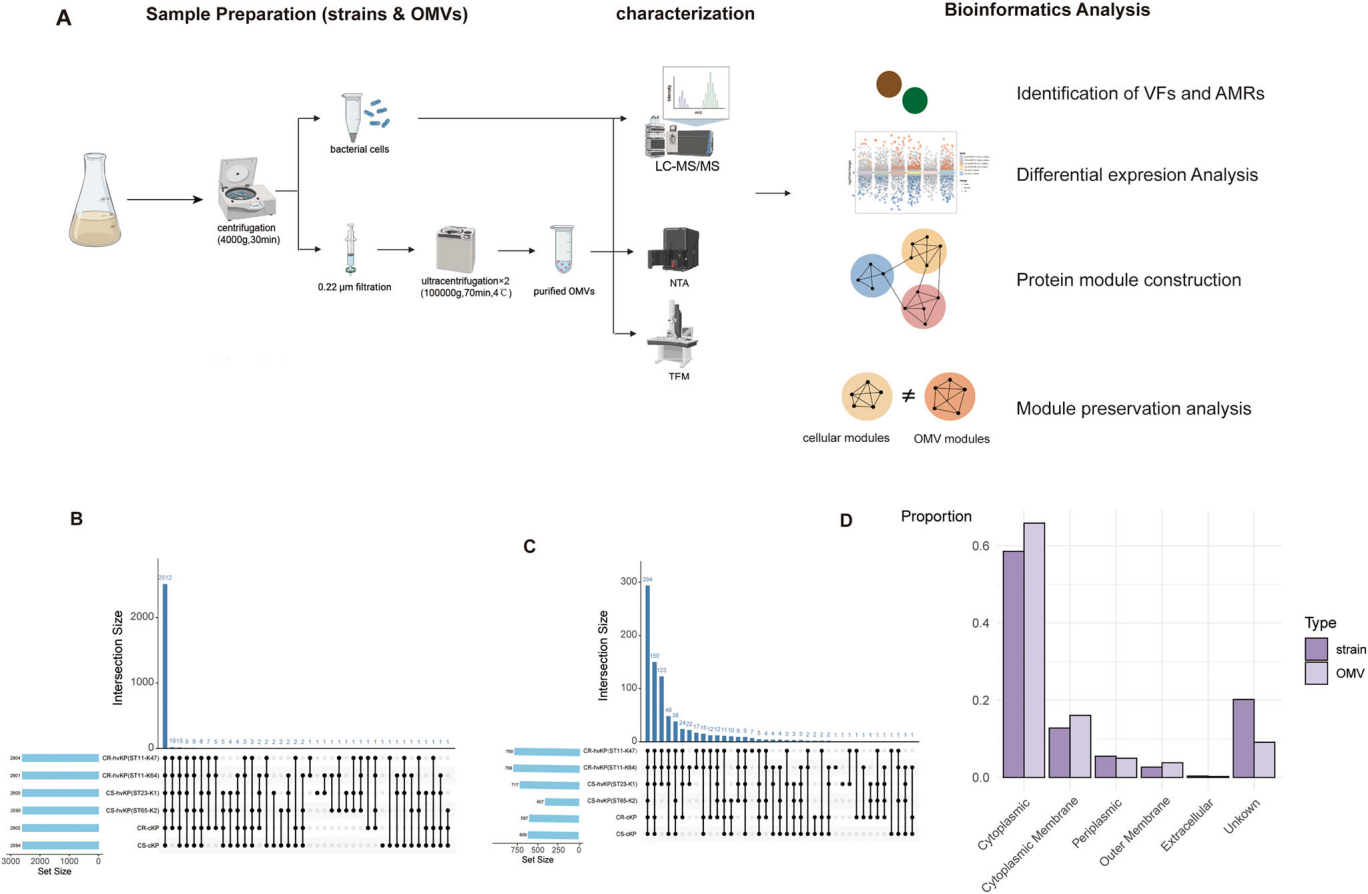
**Overview of cellular and OMV proteomes in *K. pneumoniae*. (A)**Workflow of sample preparation, characterisation, and bioinformatics analysis of *K. pneumoniae* strains and OMVs. Figure created with BioRender.com. **(B, C)** UpSet plots displaying the number and overlap of identified proteins across six *K. pneumoniae* groups (ST11‐K47, ST11‐K64, ST23‐K1, ST65‐K2, CR‐cKP, and CS‐cKP) in strains (B) and OMVs (C)**. (D)** Subcellular localisation prediction of identified proteins in strains and OMVs based on PSORTb analysis.

The sample preparation and analysis strategies are illustrated in Figure 1A, as confirmed by TEM and NTA (Figure S1A,B), which demonstrated that the protocol effectively isolates OMVs.

We compared the proteomic profiles of strains and OMVs across six groups. The number of identified proteins was comparable across strains (Figure 1B), whereas OMV samples showed considerable variation, ranging from 407 (ST65‐K2) to 798 (ST11‐K64) (Figure 1C). Subcellular localisation prediction using PSORTb showed that cytoplasmic proteins represented the largest fraction in both strains (58.6%) and OMVs (65.9%), followed by cytoplasmic membrane proteins (12.8% and 16.1%, respectively) (Figure 1D; Table S1). Overall, nearly 80% of OMV proteins originated from the cytoplasm and cytoplasmic membrane, consistent with previous studies (Dell'Annunziata et al. 2021; Hussein et al. 2023).

### 3.2 Expression Characteristics of Virulence Factors and Antimicrobial Resistance Proteins

**FIGURE 2:**
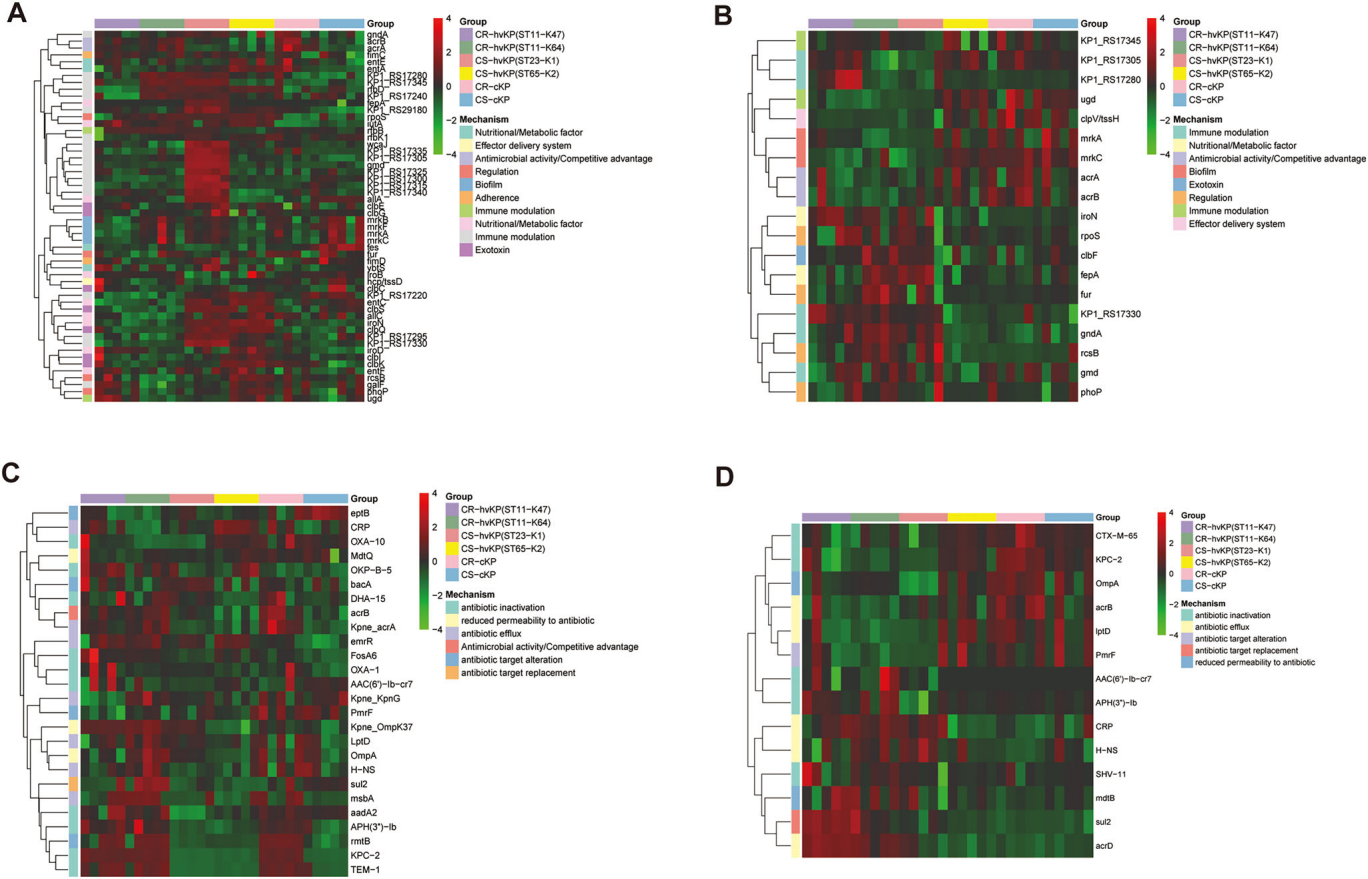
**Identification of VFs and AMRs. (A, B)** Heatmaps showing the expression profiles of VFs in strains (A) and OMVs (B). Color intensity indicates normalised protein abundance, with red for high and green for low expression. **(C,D)** Heatmaps displaying the expression profiles of AMRs in strains (C) and OMVs (D). Abundance values are z‐score normalised, where red indicates higher and blue indicates lower relative expression.

We next examined VFs and AMRs. Pairwise comparisons revealed that CS‐hvKP (ST23‐K1) and CS‐hvKP (ST65‐K2) strains contained significantly higher overall levels of VFs compared to others (Figures 2A and S2A). In particular, siderophore systems and allantoin utilisation proteins showed significantly higher abundance, including IroN/IroD (salmochelin), EntA/EntC(enterobactin), IutA (aerobactin), and AllA (allantoin utilisation), as well as colibactin (ClbK, ClbQ, ClbS). Additionally, CS‐hvKP (ST23‐K1) showed significantly elevated levels of capsule/LPS‐associated proteins such as Gmd, KP1_RS17315, and KP1_RS17325, along with the enterobactin receptor FepA. Notably, several VFs such as GndA, KP1_RS17330 were not only detected but also highly enriched in the OMVs of CR‐hv(ST11‐K47 and ST11‐K64) (Figures 2B and S2A). CRKP strains (ST11‐K47, ST11‐K64 and CR‐c) showed higher levels of β‐lactamases (e.g., KPC‐2, TEM‐1), the 16S rRNA methylase RmtB, the AcrB efflux system, and the aminoglycoside‐modifying enzyme APH(3″)‐Ib (Figures 2C and S1B). Sul2 (sulfonamide resistance), and AcrD (efflux) displayed significantly higher abundance in OMVs derived from CR‐hvKP strains (ST11‐K47 and ST11‐K64) compared with other groups (Figures 2D and S2B).

### 3.3 Highly Expressed Proteins Across Groups and Functional Enrichment

**FIGURE 3:**
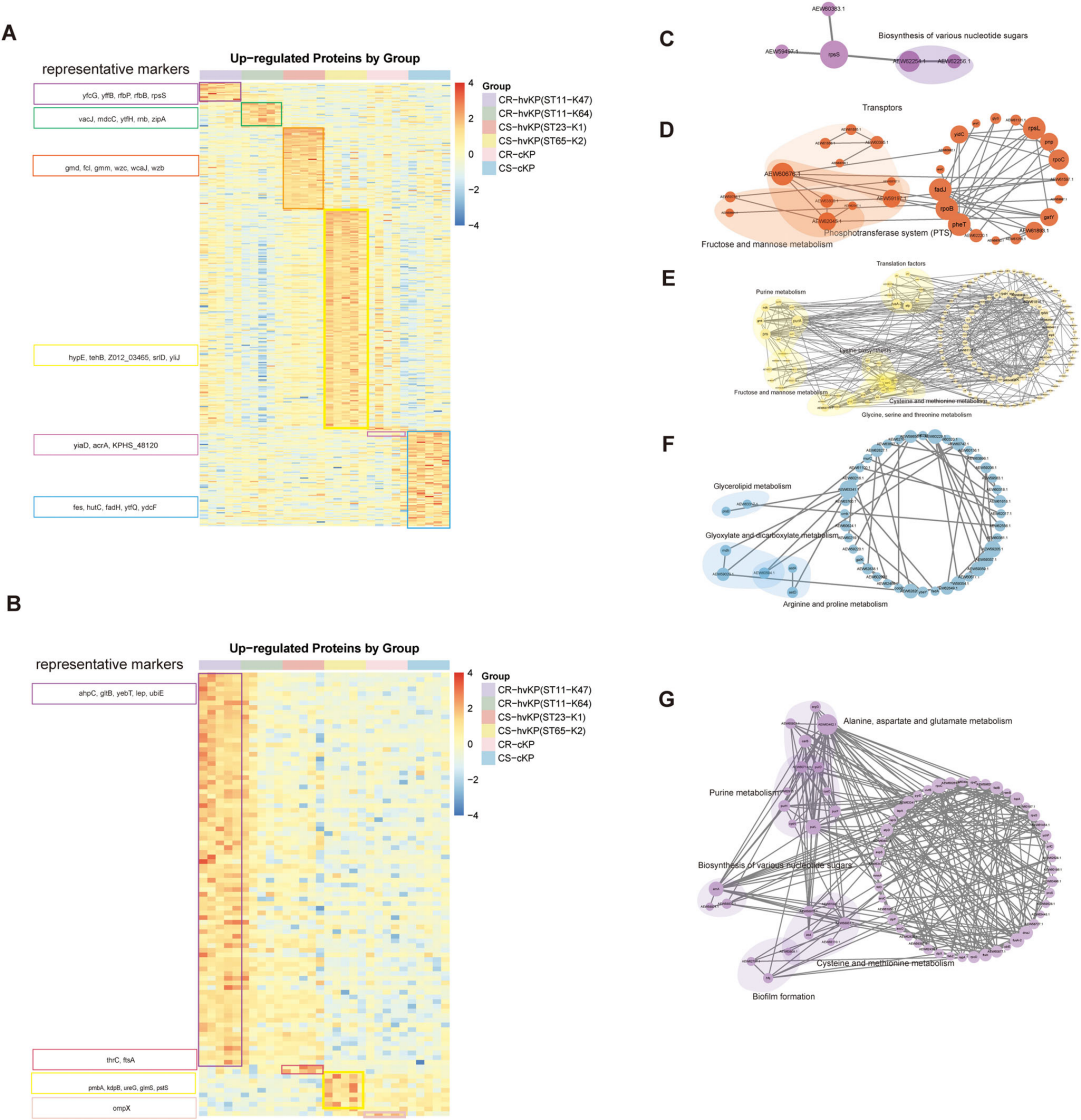
**Group‐specific upregulated proteins and their functional networks in *K. pneumoniae* strains and OMVs. (A, B)** Heatmaps showing significantly upregulated proteins in each group (ST11‐K47, ST11‐K64, ST23‐K1, ST65‐K2, CR‐c, CS‐c) compared to the others in strains (A) and OMVs (B). The right panel lists representative marker proteins with the highest statistical significance in each group. **(C–G)** PPI networks of highly expressed proteins in strains (C–F) and OMVs (G). The size of the node represents the number of interacting proteins. Proteins not contributing to the PPI network were filtered out. **(C)** ST11‐K47: Biosynthesis of various nucleotide sugars. **(D)** ST23‐K1: fructose and mannose metabolism, transporters, phosphotransferase system (PTS), biofilm formation. **(E)** ST65‐K2: translation factors, glycine, serine, and threonine metabolism, cysteine and methionine metabolism, fructose and mannose metabolism, purine metabolism, lysine biosynthesis, arginine and proline metabolism. **(F)** CS‐cKP: glycerolipid metabolism, glyoxylate and dicarboxylate metabolism, arginine and proline metabolism. **(G)** ST11‐K47: alanine, aspartate and glutamate metabolism, purine metabolism, cysteine and methionine metabolism, biosynthesis of various nucleotide sugars, and biofilm formation.

We performed differentially expressed protein (DEP) analysis across the six groups (Figure 3A,B; Table S2) and conducted functional enrichment and protein‐protein interaction (PPI) network analyses on the upregulated proteins in each group (Figure 3C–3G; Table S3). Proteins not contributing to the PPI network were filtered out.

In strains, CR‐hvKP (ST11‐K47) showed significant enrichment in pathways related to nucleotide sugar biosynthesis (Figure 3C), with key proteins linked to LPS assembly (RfbP, RfbB). CS‐hvKP (ST23‐K1 and ST65‐K2) could be distinguished by their enrichment in fructose and mannose metabolism(Figure 3D,E), which supply essential precursors for capsular polysaccharide (CPS) biosynthesis (Sfar et al. 2023). In addition, CS‐hvKP (ST23‐K1) was enriched in capsule biosynthesis, expressing capsule assembly proteins (Wzc/Wzb/Wza) and sugar precursor enzymes (Gmd, Fcl) (Figure 3A). CS‐hvKP (ST65‐K2) upregulated sorbitol metabolism proteins SrlB and SrlD (Figure 3A). In the CS‐cKP group, upregulated proteins were enriched in glycerolipid metabolism, glyoxylate/dicarboxylate metabolism and Arginine and proline metabolism (Figure 3F).

The enrichment profiles of OMVs were distinct from those of strains and also varied among groups. In CR‐hvKP (ST11‐K47) OMVs, upregulated proteins were associated with amino acid metabolism and biofilm formation (Figure 3B,G). The upregulated proteins included stress‐response proteins (AhpC, DegS), transcriptional and translational regulators (LepA, RpoD, RapA, Hfq), metabolic enzymes (GltB, UbiE, PurH, FolK, Asd, CysD, TyrB), etc. CS‐hvKP (ST23‐K1) OMVs showed increased abundance of ThrC and FtsA. CS‐hvKP (ST65‐K2) OMVs were characterised by elevated levels of PmbA, KdpB, and UreG. In CR‐cKP OMVs, OmpX was upregulated.

### 3.4 Weighted Co‐Expression Network Analysis Identifies Modules Associated With Strain Characteristics

**FIGURE 4:**
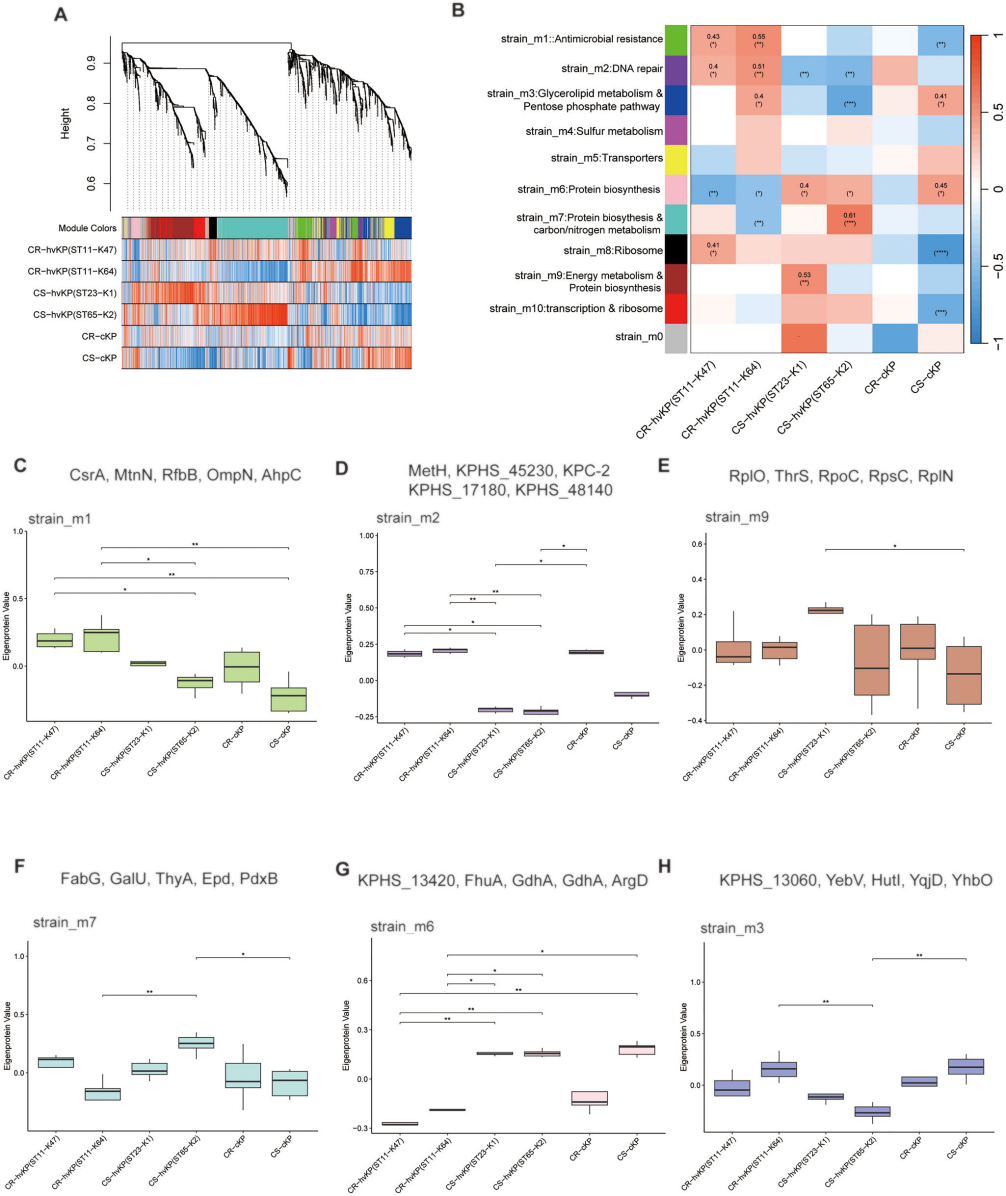
**Co‐expression network construction in strains. (A)** Dendrogram of protein clustering based on topological overlap, with identified modules coloured beneath. **(B)** Heatmap showing the correlations between cellular MEs and six groups (ST11‐K47, ST11‐K64, ST23‐K1, ST65‐K2, CR‐c, CS‐c). Each cell represents the Pearson correlation coefficient between ME and the indicated group. The colour scale indicates the direction and strength of the correlation (red, positive; blue, negative). **(C–H)** Boxplots of ME expression levels across group‐associated modules. Statistical significance was assessed using the Kruskal–Wallis test followed by Dunn's post hoc test for multiple comparisons; *P < 0.05, **P < 0.01, ***P < 0.001, ****P < 0.0001. For each module, the top five proteins with the highest KME are listed above the corresponding boxplot.

To understand the co‐expression relationships between proteins at a systems level, we performed weighted co‐expression network analysis (WGCNA) (Wiśniewski et al. 2009). This unsupervised and unbiased analysis identified distinct co‐expression modules corresponding to clusters of correlated proteins (Figure 4A). Soft‐thresholding analysis showed that a β value of 5 achieved an approximate scale‐free topology while retaining acceptable mean connectivity (Figure S3A). We next assessed the internal robustness of the strain network using split‐sample module preservation analysis. All identified strain modules showed at least moderate preservation in the test subset (Zsummary > 2), and six modules showed strong preservation (Zsummary > 10), supporting the internal stability of the cellular co‐expression network (Figure S3B). Using dynamic tree cutting and merging of similar modules (correlation threshold = 0.75), a total of ten distinct modules were identified, each represented by a unique color (Figure 4B). Since each co‐expression module groups together correlated proteins, each module can be represented by a single representative expression profile called module eigenprotein (ME) (Langfelder and Horvath 2007). After module identification, we analysed the enrichment of proteins in each co‐expression module (Table S4). Several modules showed strong associations with specific groups. The strain_m1 and strain_m2 modules, which are enriched for proteins involved in antimicrobial resistance and DNA repair, were upregulated in CR‐hvKP (ST11‐K47 and ST11‐K64) (Figure 4B–4D). Within this module, key AMRs such as KPC‐2, TEM‐1, and RmtB were identified (Figure S4A). The strain_m9 module, associated with protein biosynthesis and energy metabolism, was upregulated in CS‐hvKP (ST23‐K1) (Figure 4B,E). This module was also enriched for VFs related to capsule biosynthesis (Figure S4B). Similarly, the strain_m7 module, enriched in protein biosynthesis and carbon/nitrogen metabolism, was upregulated in another hypervirulent strain, CS‐hvKP (ST65‐K2) (Figure 4B,F), and contained numerous VFs (Figure S4B). Strain_m3 and Strain_m6 were enriched in lipid metabolism and protein biosynthesis, respectively, and both modules exhibited a positive correlation with CS‐cKP (Figure 4B,G,H).

Proteins with the highest connectivity (kME) within each module, designated as hub proteins, are shown in Figures 4C–H and listed in Table S5. Representative hub proteins were CsrA, MtnN, RfbB, OmpN, and AhpC in strain_m1; MetH, KPHS_45230, KPC‐2, KPHS_17180, and KPHS_48140 in strain_m2; RplO, ThrS, RpoC, RpsC, and RplN in strain_m9; and additional hub proteins in the remaining modules are provided in Table S5.

### 3.5 Weighted Co‐Expression Network Analysis Reveals Modules Distinct from Strains and Reflecting OMV Characteristics

We next constructed a weighted co‐expression network for the OMV proteome. Soft‐thresholding analysis indicated that a β value of 8 provided an approximate scale‐free topology with acceptable mean connectivity (Figure S3C). Split‐sample preservation analysis further showed that the OMV modules were overall preserved in the test subset, indicating acceptable internal stability of the OMV co‐expression network (Figure S3D).

**FIGURE 5:**
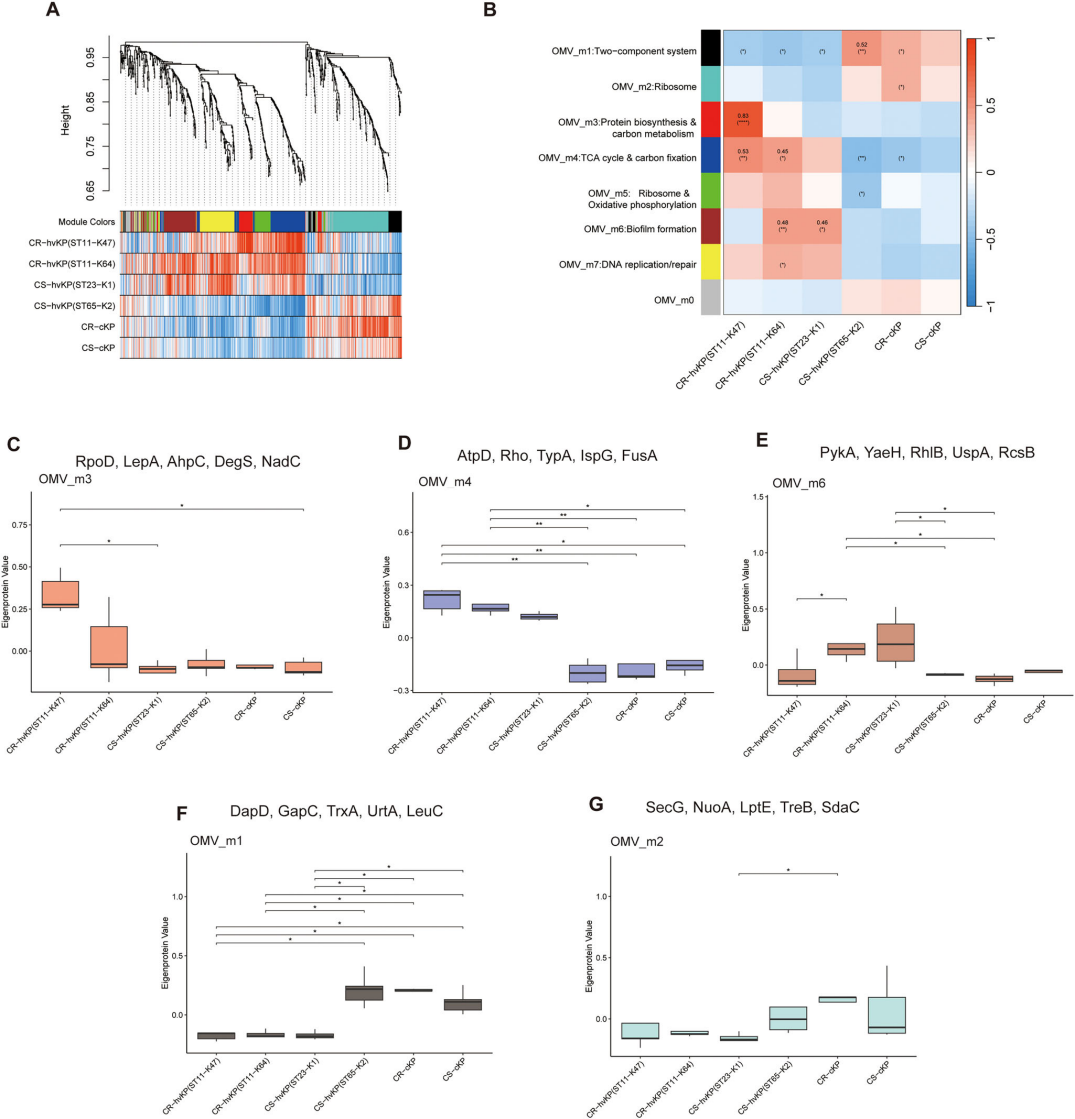
**Co‐expression network construction in OMVs. (A)** Dendrogram of protein clustering based on topological overlap, with identified modules coloured beneath. **(B)** Heatmap showing the correlations between MEs and six groups (ST11‐K47, ST11‐K64, ST23‐K1, ST65‐K2, CR‐c, CS‐c) in OMVs. Each cell represents the Pearson correlation coefficient between ME and the indicated group. The colour scale indicates the direction and strength of the correlation (red, positive; blue, negative). **(C–G)** Boxplots of ME expression levels across group‐associated modules. Statistical significance was assessed using the Kruskal–Wallis test followed by Dunn's post hoc test for multiple comparisons; *P < 0.05, **P < 0.01, ***P < 0.001, ****P < 0.0001. For each module, the top five proteins with the highest KME are listed above the corresponding boxplot.

Based on this network, dynamic tree cutting identified seven distinct co‐expression modules (Figure 5A). The expression of these module eigenproteins was associated with specific groups (Figure 5B). The OMV_m3 and OMV_m4 modules, upregulated in CR‐hvKP (ST11‐K47), were enriched in carbon metabolism/protein biosynthesis and TCA cycle (Figures 5B–5D). Hub proteins included RpoD, LepA, AhpC, DegS and NadC in OMV_m3, and AtpD, Rho, TypA, IspG and FusA in OMV_m4. The OMV_m6 module, associated with CR‐hvKP (ST11‐K64) and CS‐hvKP (ST23‐K1) (Figure 5B,E), was linked to biofilm formation, with hub proteins PykA, YaeH, RhlB, UspA and RcsB. The OMV_m1 module, upregulated in CS‐hvKP (ST65‐K2), was enriched in two−component system, with hub proteins such as DapD, GapC, TrxA, UrtA and LeuC (Figure 5B,F). The OMV_m2 module, upregulated in CR‐cKP, was enriched in ribosome, with hub proteins such as SecG, NuoA, LptE, TreB and SdaC (Figure 5B,G). The VFs and AMRs of each module are shown in Figure S5A,B.

We next integrated the findings from differential expression and co‐expression network analyses. Proteins that were significantly upregulated in a particular group were consistently clustered into co‐expression modules that also showed strong associations with the same groups (Figures S3C and S4C). This agreement between the two analytical approaches suggests that each group possesses a unique molecular signature, evident not only in individual protein expression levels but also in the co‐expressed protein network.

### 3.6 Module Preservation Analysis Demonstrates Limited Similarity Between Strains and OMVs

Previous studies have focused on either bacterial expression profiles or OMVs, but none have systematically compared co‐expression networks between OMVs and their corresponding source strains. To address this gap, we performed a module preservation analysis to assess whether the co‐expression modules identified in OMVs were also preserved in strains (Zhou et al. 2023).

We found that most OMV modules showed no preservation in the cellular network (Zsummary < 2) (Figure 5A). These non‐preserved modules included the CR‐hvKP (ST11‐K47)‐associated OMV_m1, the CR‐hvKP (ST11‐K47 and ST11‐K64)‐associated OMV_m4 modules, the CS‐hvKP (ST23‐K1)‐associated OMV_m1 module, and the CR‐cKP‐associated OMV_m2 module. The lack of preservation of most OMV modules indicates that the co‐expression relationships among proteins in OMVs are largely distinct from those in strains. To quantify the concordance between lineage‐associated changes in cellular and OMV proteomes, we calculated Spearman rank correlations between cellular and OMV log2FC values for proteins detected in both fractions (Figure 5B). Across all six lineage comparisons, the correlations were weak, with ρ values ranging from −0.081 to 0.142, and even the highest significant correlation explained less than 2% of the variance. This is consistent with the module preservation analysis, indicating that protein levels in OMVs are not simply a reflection of their cellular abundance. Together, these findings highlight that the functional organisation of proteins in OMVs is largely distinct from that in their source strains.

**FIGURE 6:**
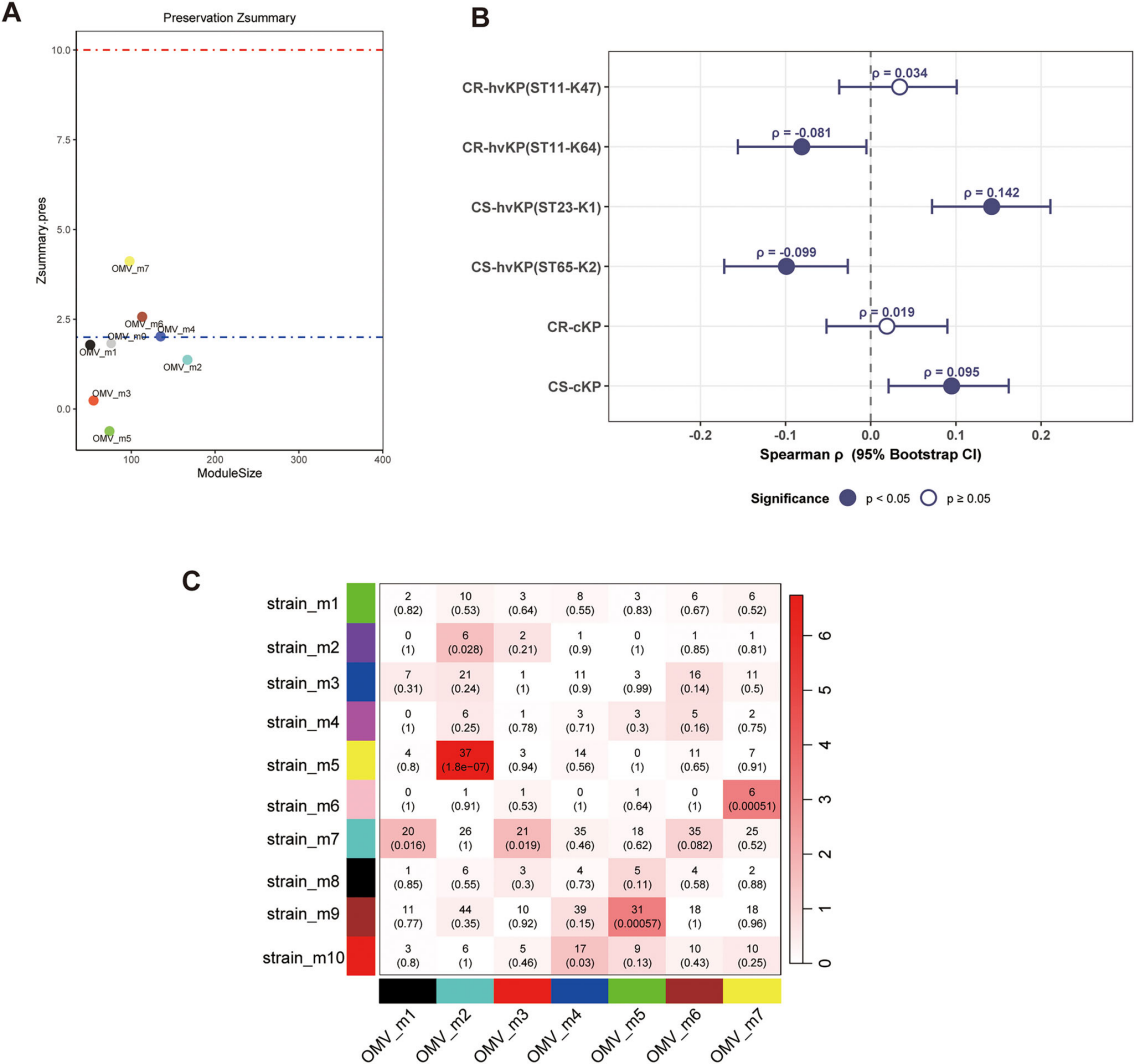
**Module preservation and cross‐compartment correspondence between strains and OMVs. (A)** Preservation Zsummary statistics of OMV modules in strain data. Each point represents a module. Point colour reflects the module colour as used in Figure 5A; Blue and red lines depict the rough thresholds for moderate (*Z* = 2) and strong (*Z* = 10) evidence of module preservation. **(B)** Correlation of protein abundance changes between cellular and OMV proteomes. Each point represents the Spearman rank correlation coefficient (ρ) between cellular and OMV log_2_ fold‐change values for proteins detected in both fractions in a given lineage comparison. Log_2_ fold change was calculated as each focal lineage versus all other lineages combined. Horizontal bars indicate 95% bootstrap confidence intervals estimated from 1,000 resamples. Filled circles denote correlations with *p* < 0.05, whereas open circles indicate *p* ≥ 0.05. **(C)** Heatmap illustrating the overlap of proteins between OMV and cellular co‐expression modules. The x‐axis and y‐axis represent modules derived from OMVs and strains, respectively. Each cell contains the number of shared proteins and the corresponding hypergeometric *p*‐value. Colour intensity reflects statistical significance as measured by −log10 (*p*‐value).

To provide a more intuitive view of cross‐compartment preservation, we further examined the overlap of proteins between OMV and cellular co‐expression modules (Figure 6C). The heatmap summarises pairwise module overlaps, with each cell showing the number of shared proteins and the corresponding hypergeometric *p* value. Overall, only a limited number of module pairs showed appreciable overlap, consistent with the weak cross‐compartment preservation observed above.

## 4 Discussion

We present the first comprehensive proteomic analysis that jointly examines *K. pneumoniae* strains and their OMVs across major clinical lineages. Through detailed protein profiling, our study not only confirms the pivotal role of OMVs in transmitting VFs and AMRs but also reveals substantial differences in proteomic composition between cellular and OMV fractions across lineages. This integrative approach offers a novel molecular perspective on the pathogenic mechanisms of *K. pneumoniae*.

First, our study clearly delineates the fundamental molecular divergence between CRKP and HvKP. In the cellular proteome, CRKP significantly upregulated the strain_m1 and strain_m2 modules, which are enriched in antimicrobial resistance and DNA repair, suggesting that CRKP relies on complex regulatory networks to maintain genomic stability and adapt under antibiotic stress (Song et al. 2024; Molan and Žgur Bertok 2022; Chittò et al. 2020; Brand et al. 2025). A particularly noteworthy observation is that, compared with classical hypervirulent strains (ST23‐K1 and ST65‐K2), CR‐hvKP (ST11‐K47 and ST11‐K64) shows overall lower cellular expression levels of VFs. This likely reflects the fitness cost imposed by strong antibiotic stress, in which bacteria redirect cellular resources (e.g., energy, amino acids) toward the synthesis of AMRs (such as KPC‐2, RmtB, and the AcrAB efflux pump), thereby reducing investment in VF production (Zhou et al. 2023; Tian et al. 2022).

In contrast, CS‐hvKP (ST23‐K1 and ST65‐K2) diverts resources toward biosynthesis and metabolism, leveraging their robust protein synthesis capacity to produce VFs. Notably, CS‐hvKP (ST23‐K1) exhibited significantly higher expression of capsule/LPS synthesis proteins (e.g., Wzc, Wza and G;md). Gmd is essential for generating GDP‐mannose, the key precursor required for K1 capsule formation. This elevated expression likely explains the potent anti‐phagocytic property and high invasiveness associated with the K1 serotype, which is clinically linked to a heightened risk of liver abscesses (Chen et al. 2022; Pan et al. 2015). Conversely, ST65‐K2 appears to employ a distinct strategy, enriching its OMVs with two‐component system proteins. This enrichment may bolster nutrient acquisition, environmental sensing, and virulence regulation, thereby supporting host adaptation and facilitating invasive infections across diverse tissues (McMillan and Kuehn 2023; Li et al. 2016; Guo et al. 2017).

Secondly, this study provides detailed evidence characterising OMVs as functionally specialised vesicles. Although OMV proteins are largely derived from the cytoplasm and cytoplasmic membrane, their co‐expression networks differ significantly from those of the parent strains. Correlation analysis demonstrates a weak correlation between cellular and OMV proteins. Most importantly, module preservation analysis identified most OMV‐specific modules that were not preserved in cellular networks (Zsummary < 2). Thus, OMVs are not passive extensions of the cellular proteome but are more likely to represent reconfigured functional units (Lucena et al. 2023; Orench‐Rivera and Kuehn 2021; Cahill et al. 2015). During infection, they may perform an indispensable, non‐redundant role (Yao et al. 2023; Dell'Annunziata et al. 2024) in pathogenicity. Our identification of specific protein signatures in OMVs highlights their diagnostic potential. OMVs are stable, abundant in body fluids during infection, and protect their protein cargo from degradation. These features make them suitable for liquid biopsy. Such an approach could support strain typing and early diagnosis, while offering faster and more sensitive results than traditional culture‐based methods.

This study has several limitations. First, OMVs were isolated using differential ultracentrifugation without density gradient purification or protease protection assays; thus, the samples may contain residual non‐OMV proteins or cellular debris. Second, the use of different MS acquisition modes for strains and OMVs may affect cross‐compartment comparisons. Third, protein identification relied on a reference genome database, which may not fully capture lineage‐specific accessory proteins. Fourth, technical replicates were not performed for each isolate, which may affect the precision of quantitative estimates. Finally, this study was primarily conducted under in vitro culture conditions, and the mechanisms of OMV cargo packaging may differ in the complex host environment (Orench‐Rivera and Kuehn 2021). In addition, proteomics has detection limits, meaning some low‐abundance proteins may not be captured (Li and Smyth 2023; Li et al. 2021).

Future studies should address these limitations by incorporating density gradient purification, unified data acquisition platforms, and lineage‐specific proteogenomic databases to improve data quality and cross‐group comparability. Functional validation of hub proteins identified in this study, together with exploration of OMV‐based liquid biopsy strategies and targeted therapeutic interventions, will be critical for translating these findings into actionable approaches against high‐risk *K. pneumoniae* clones. In summary, this study advances our understanding of the molecular strategies underlying *K. pneumoniae* pathogenicity and highlights the specialised contribution of OMVs to resistance and virulence, providing a foundation for future mechanistic and translational research.

## Author Contributions

**Zongping Li**: writing – original draft, visualization, methodology, formal analysis. **Jingyuan Xi**: investigation, data curation, writing – review and editing, methodology, conceptualization. **Xinmiao Jia**: supervision, methodology, conceptualization. **Yangzhige He**: methodology, supervision, conceptualization. **Guibin Wang**: data curation, investigation, methodology. **Shiyu Chen**: writing – review and editing. **Xiaobing Chu**: writing – review and editing. **Qian Zhang**: writing – review and editing. **Ying Zhu**: writing – review and editing. **Wei Yu**: writing – review and editing. **Peiyao Jia**: writing – review and editing. **Xiaoyu Liu**: writing – review and editing. **Qiwen Yang**: conceptualization, writing – review and editing, project administration, funding acquisition, supervision, resources.

## Funding

This work was supported by CAMS Innovation Fund for Medical Sciences (CIFMS) (2025‐I2M‐XHJC‐004), CAMS Innovation Fund for Medical Sciences (2025‐I2M‐KJ‐001), National Science and Technology Major Project (2025ZD01903400 and 2024ZD0532804), the Fundamental Research Funds for the Central Universities, Peking Union Medical College (3332024203), the Peking Union Medical College Hospital Talent Cultivation Program (Category D) (UHB12054), CAMS Innovation Fund for Medical Sciences (CIFMS) (2025‐I2M‐XHCL‐013). The funders had no role in the study design, data collection and analysis, decision to publish, or preparation of the manuscript.

## Conflicts of Interest

The authors declare no conflicts of interest.

## Supporting information




**Figure S1. Characterisation of purified OMVs from *K. pneumoniae*. (A)** Negative‐staining TEM image of purified OMVs, showing spherical vesicles with a bilayered membrane structure. Scale bar, 200 nm. **(B)** Representative NTA profile of purified OMVs, showing a mean particle size of 113.2 nm, with most vesicles distributed between 50 and 200 nm.

**Figure S2. Comparative analysis of VFs and AMRs in *K. pneumoniae*. (A)** Heatmap of VFs from strains (top) and OMVs (bottom) across pairwise strain comparisons. **(B)** Heatmap of AMRs from strains (top) and OMVs (bottom). Colors show log2 fold change (logFC) values (blue, down; red, up). Statistical significance was assessed by limma with adjusted *p‐values* (*P < 0.05, **P < 0.01, ***P < 0.001, ****P < 0.0001).

**Figure S3. Soft‐threshold selection and split‐sample module preservation analysis for the strains and OMV co‐expression networks. (A, C)** Soft‐threshold selection for the strains (A) and OMV (C) datasets. The scale‐free topology fit index and mean connectivity were evaluated across candidate soft‐thresholding powers; *β* = 5 was selected for the strain dataset and *β* = 8 for the OMV dataset. **(B, D)** Split‐sample module preservation analysis for the strains (B) and OMV (D) networks. Each point represents one module, plotted by Zsummary against log10‐transformed module size. Dashed lines indicate the conventional thresholds for moderate preservation (Zsummary = 2) and strong preservation (Zsummary = 10). These analyses were used to assess the internal robustness of the co‐expression networks under sample resampling.

**Figure S4. Distribution of AMRs, VFs and upregulated proteins across cellular modules. (A, B)** Chord diagram shows the distribution of AMRs (A) and VFs (B) across WGCNA modules. **(C)** Distribution of Upregulated Proteins Across WGCNA Modules in Each Group. Each bar represents the proportion of significantly upregulated proteins (logFC > 0.585, adjusted P < 0.05) in a specific group that is assigned to WGCNA modules. The x‐axis indicates the groups, and the y‐axis shows the proportion of upregulated proteins falling into each module.

**Figure S5. Distribution of AMRs, VFs and upregulated proteins across OMV modules. (A, B)** Chord diagram shows the distribution of AMRs (A) and VFs (B) across WGCNA modules. **(C)** Distribution of Upregulated Proteins Across WGCNA Modules in Each Group. Each bar represents the proportion of significantly upregulated proteins (logFC > 0.585, adjusted *P* < 0.05) in a specific group that is assigned to WGCNA modules. The x‐axis indicates the groups, and the y‐axis shows the proportion of upregulated proteins falling into each module.

**Supporting Information**: jex270135‐sup‐0006‐TableS1

**Supporting Information**: jex270135‐sup‐0007‐TableS2

**Supporting Information**: jex270135‐sup‐0008‐TableS3

**Supporting Information**: jex270135‐sup‐0009‐TableS4

**Supporting Information**: jex270135‐sup‐0010‐TableS5

## Data Availability

The mass spectrometry proteomics data have been deposited to the iProX database (https://www.iprox.cn) with the dataset identifier IPX0015994000.
