# Supplementary figures and images for: Comparative Proteomic Profiling of Clinical Klebsiella pneumoniae Strains and the Corresponding Outer Membrane Vesicles
Source: J Extracell Biol. 2026 Apr 14;5(4):e70135. doi: 10.1002/jex2.70135 (PMC13077552; doi:10.1002/jex2.70135)

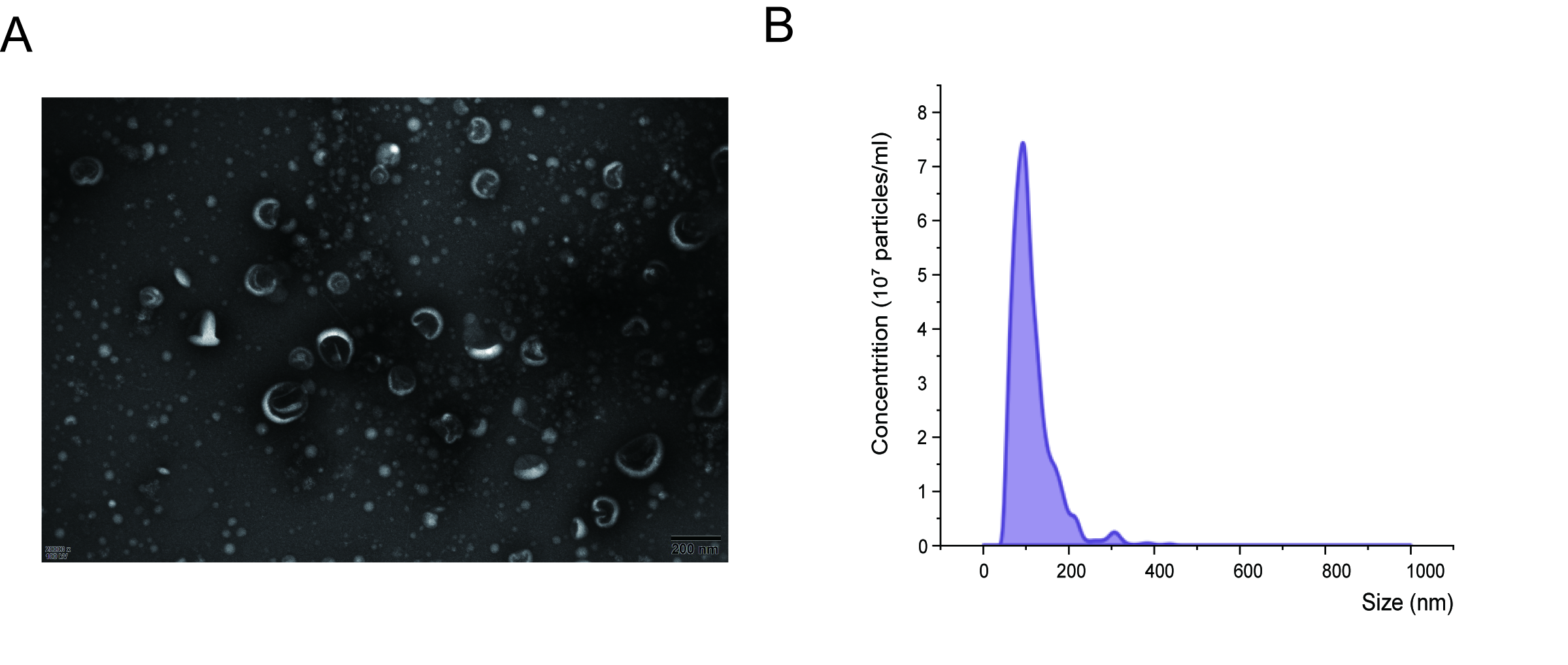

Supplement: Supplementary file 1 — Figure S1. Characterisation of purified OMVs from K. pneumoniae. (A) Negative‐staining TEM image of purified OMVs, showing spherical vesicles with a bilayered membrane structure. Scale bar, 200 nm. (B) Representative NTA profile of purified OMVs, showing a mean particle size of 113.2 nm, with most vesicles distributed between 50 and 200 nm. [file JEX2-5-e70135-s001.tif]

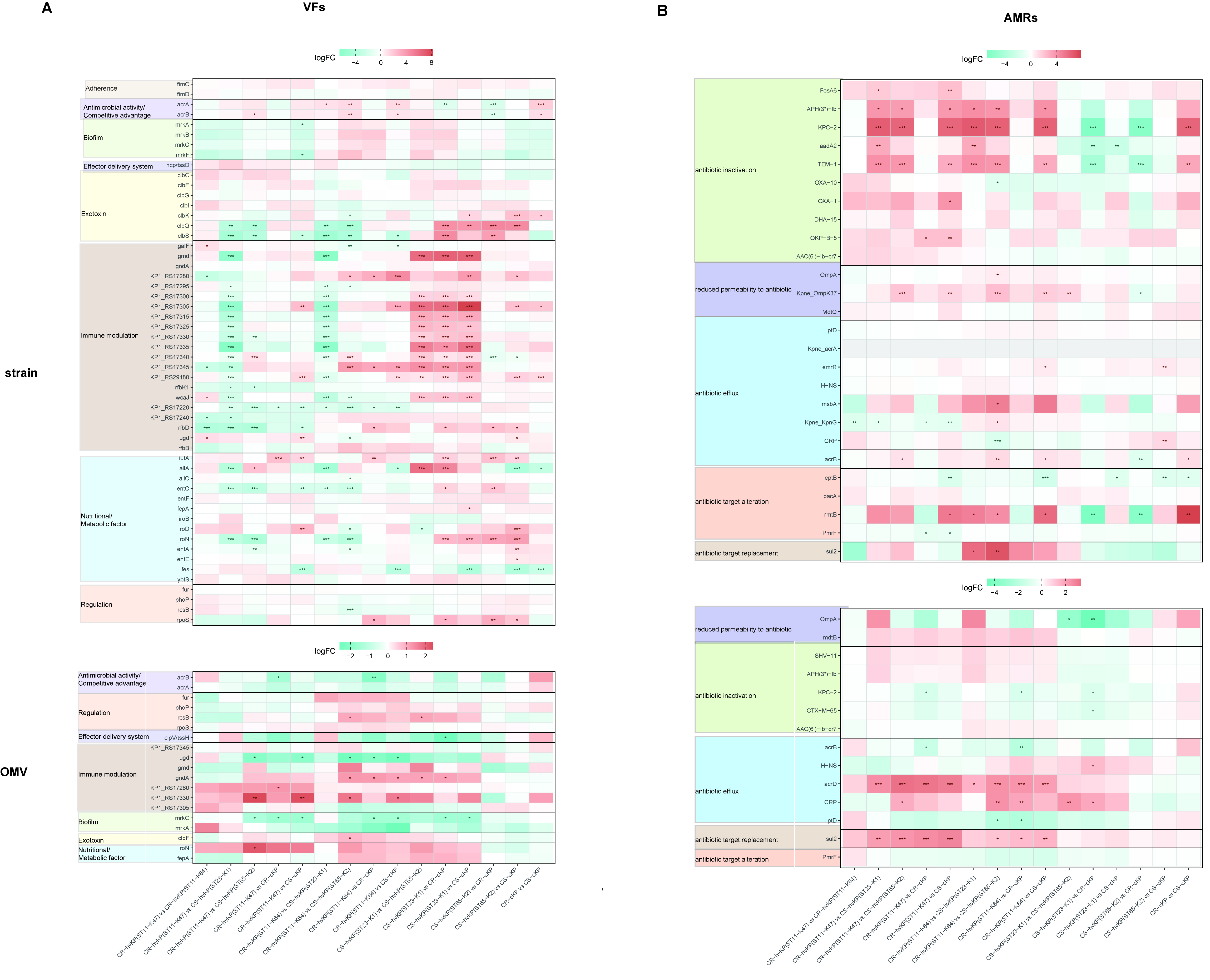

Supplement: Supplementary file 2 — Figure S2. Comparative analysis of VFs and AMRs in K. pneumoniae. (A) Heatmap of VFs from strains (top) and OMVs (bottom) across pairwise strain comparisons. (B) Heatmap of AMRs from strains (top) and OMVs (bottom). Colors show log2 fold change (logFC) values (blue, down; red, up). Statistical significance was assessed by limma with adjusted p‐values (*P < 0.05, **P < 0.01, ***P < 0.001, ****P < 0.0001). [file JEX2-5-e70135-s009.tif]

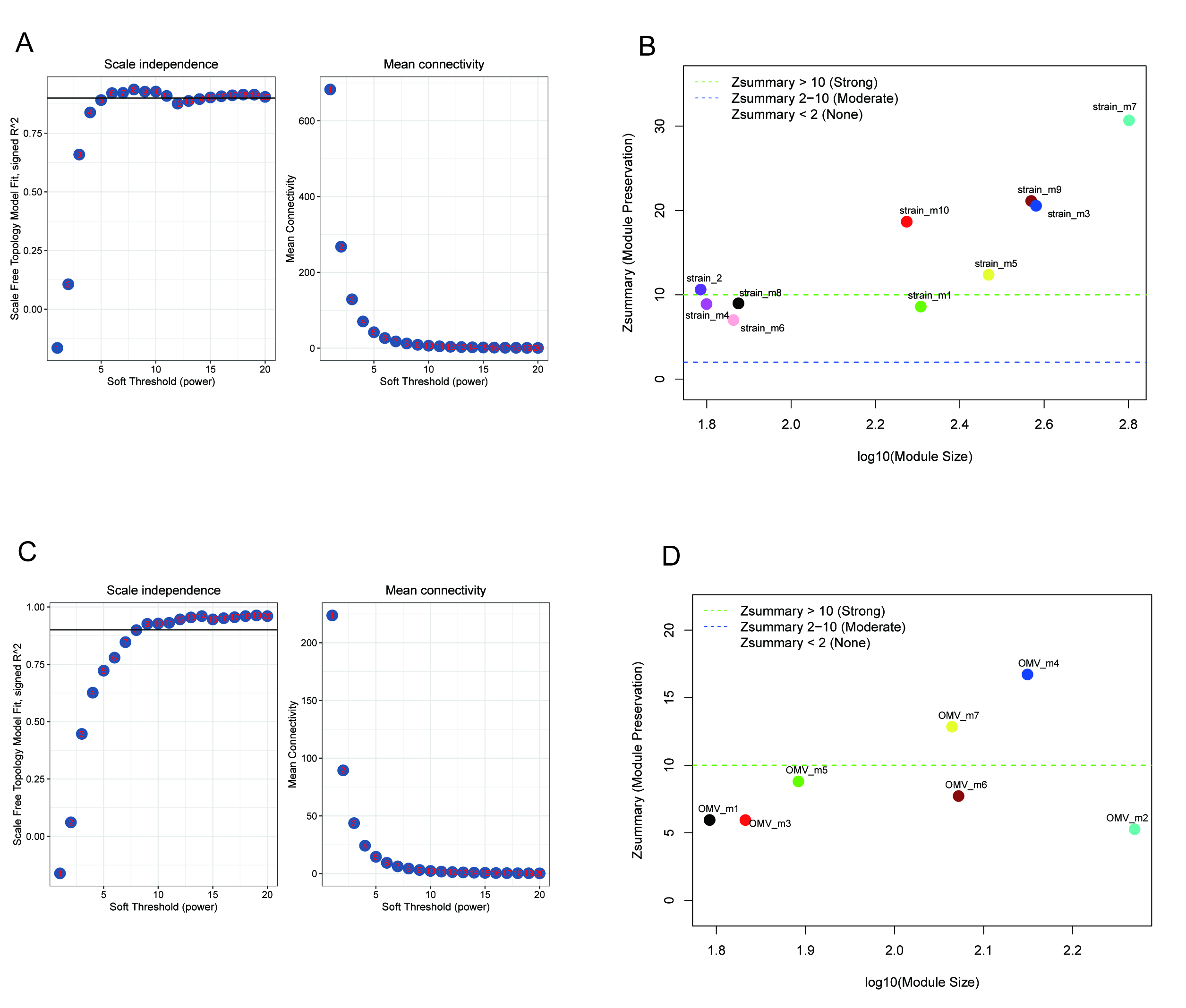

Supplement: Supplementary file 3 — Figure S3. Soft‐threshold selection and split‐sample module preservation analysis for the strains and OMV co‐expression networks. (A, C) Soft‐threshold selection for the strains (A) and OMV (C) datasets. The scale‐free topology fit index and mean connectivity were evaluated across candidate soft‐thresholding powers; β = 5 was selected for the strain dataset and β = 8 for the OMV dataset. (B, D) Split‐sample module preservation analysis for the strains (B) and OMV (D) networks. Each point represents one module, plotted by Zsummary against log10‐transformed module size. Dashed lines indicate the conventional thresholds for moderate preservation (Zsummary = 2) and strong preservation (Zsummary = 10). These analyses were used to assess the internal robustness of the co‐expression networks under sample resampling. [file JEX2-5-e70135-s005.tif]

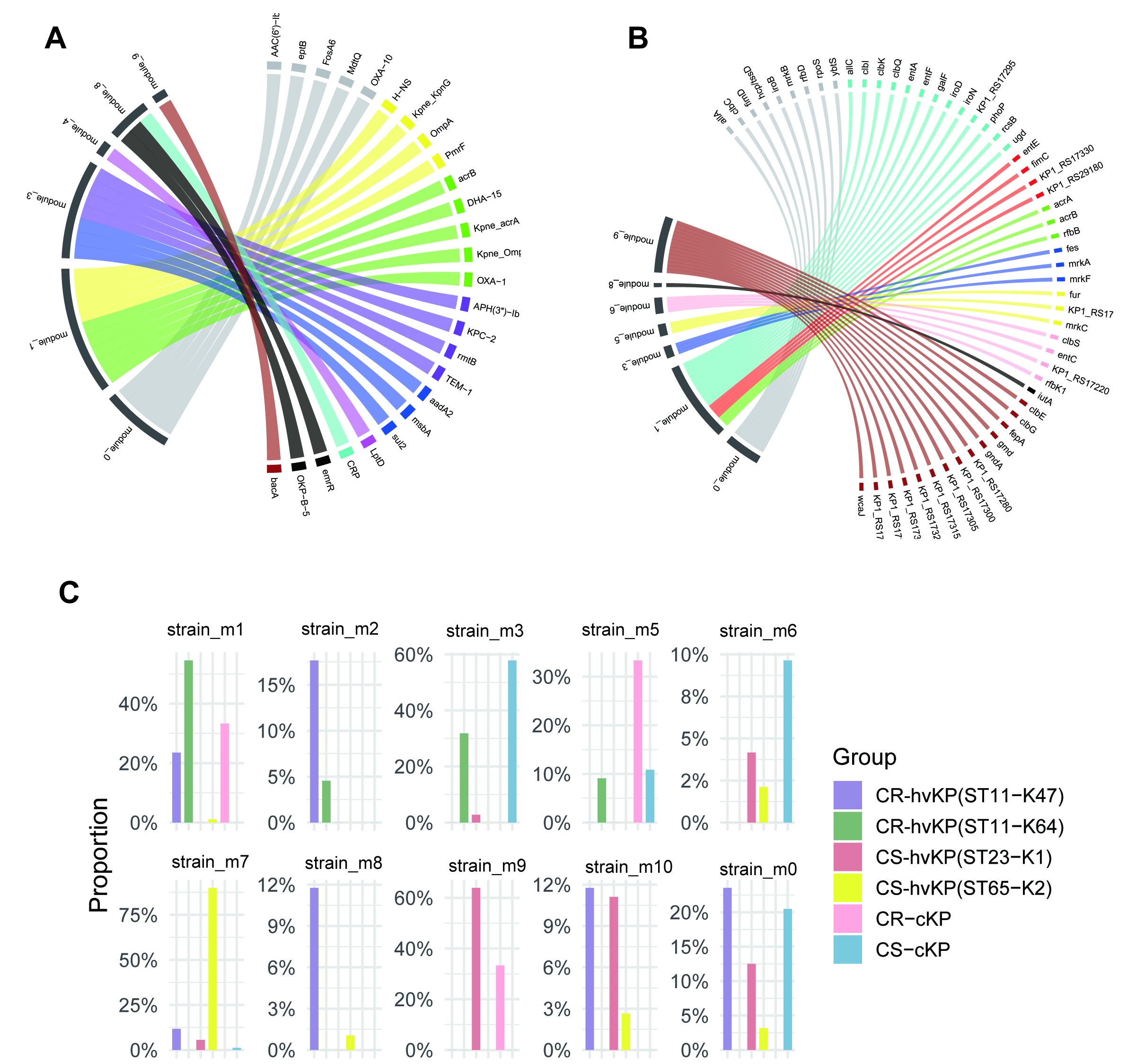

Supplement: Supplementary file 4 — Figure S4. Distribution of AMRs, VFs and upregulated proteins across cellular modules. (A, B) Chord diagram shows the distribution of AMRs (A) and VFs (B) across WGCNA modules. (C) Distribution of Upregulated Proteins Across WGCNA Modules in Each Group. Each bar represents the proportion of significantly upregulated proteins (logFC > 0.585, adjusted P < 0.05) in a specific group that is assigned to WGCNA modules. The x‐axis indicates the groups, and the y‐axis shows the proportion of upregulated proteins falling into each module. [file JEX2-5-e70135-s007.tif]

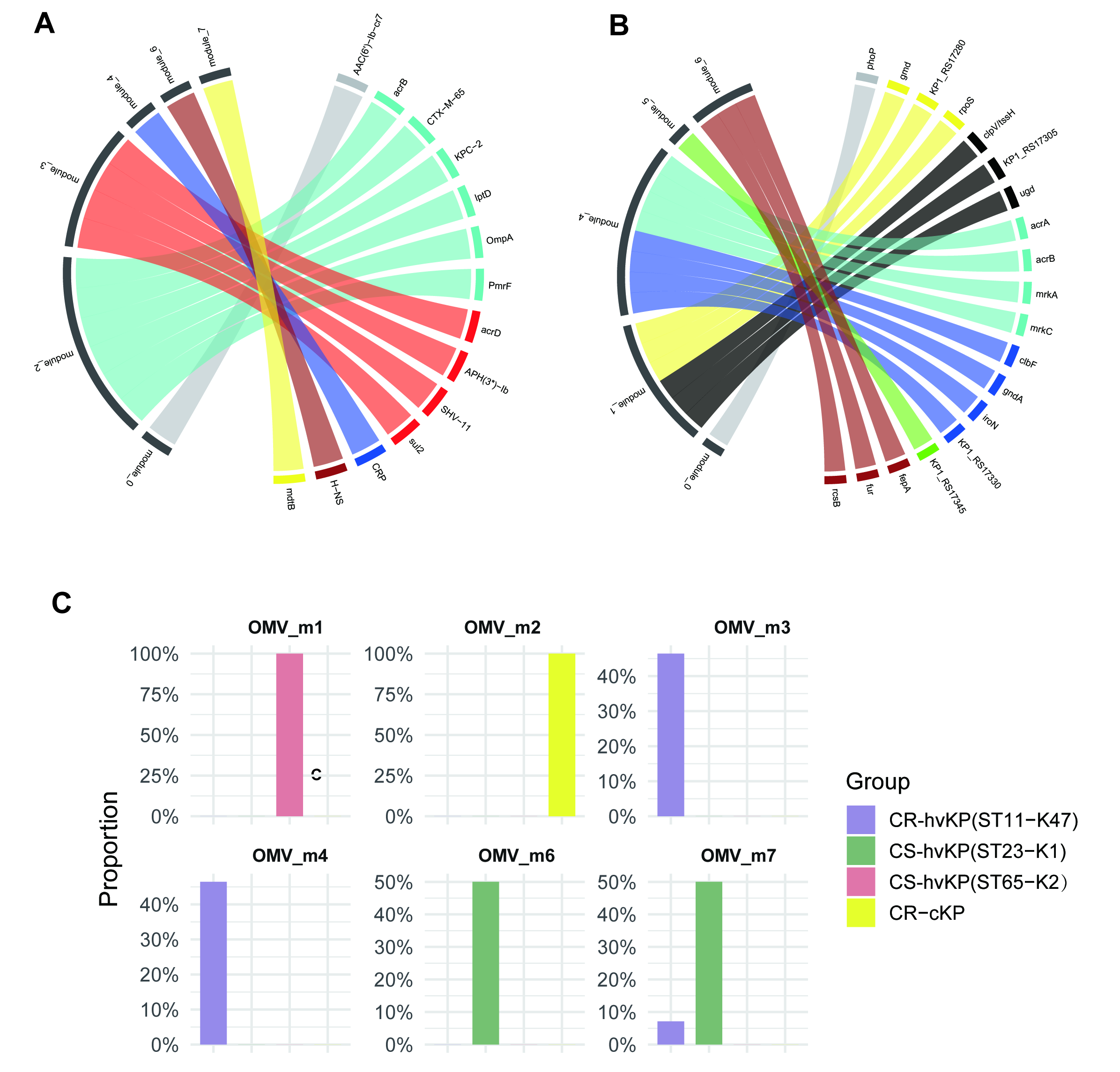

Supplement: Supplementary file 5 — Figure S5. Distribution of AMRs, VFs and upregulated proteins across OMV modules. (A, B) Chord diagram shows the distribution of AMRs (A) and VFs (B) across WGCNA modules. (C) Distribution of Upregulated Proteins Across WGCNA Modules in Each Group. Each bar represents the proportion of significantly upregulated proteins (logFC > 0.585, adjusted P < 0.05) in a specific group that is assigned to WGCNA modules. The x‐axis indicates the groups, and the y‐axis shows the proportion of upregulated proteins falling into each module. [file JEX2-5-e70135-s003.tif]
